# Supplementary material for: Mobile interventions targeting common mental disorders among pregnant and postpartum women: An equity-focused systematic review
Source: PLoS One. 2021 Oct 29;16(10):e0259474. doi: 10.1371/journal.pone.0259474 (PMC8555821; doi:10.1371/journal.pone.0259474)
Supplement: S2 File — (DOCX) [file pone.0259474.s002.docx]

**Mobile interventions targeting common mental disorders among pregnant and postpartum women: An equity-focused systematic review**

**Appendix II: PRISMA-E checklist**

Only equity-extension items not described in the PRISMA checklist were reported here

| **Checklist of Items for Reporting Equity-Focused Systematic Reviews** | | |  |
| --- | --- | --- | --- |
| **Section** | **Item** | **Extension for Equity-Focused Reviews** | **Pg #** |
| **Title** |  |  |  |
| **Title** | 1 | Identify equity as a focus of the review, if relevant, using the term equity | 1 |
| **Abstract** |  |  |  |
| **Structured summary** | 2 | State research question(s) related to health equity. | 2 |
|  | 2A | Present results of health equity analyses (e.g. subgroup analyses or meta-regression). | 2 |
|  | 2B | Describe extent and limits of applicability to disadvantaged populations of interest. | 2 |
| **Introduction** |  |  |  |
| **Rationale** | 3 | Describe assumptions about mechanism(s) by which the intervention is assumed to have an impact on health equity. | 3 |
|  | 3A | Provide the logic model/analytical framework, if done, to show the pathways through which the intervention is assumed to affect health equity and how it was developed. | Protocol |
| **Objectives** | 4 | Describe how disadvantage was defined if used as criterion in the review (e.g. for selecting studies, conducting analyses or judging applicability). | 3-4 |
|  | 4A | State the research questions being addressed with reference to health equity | 3-4 |
| **Methods** |  |  |  |
| **Eligibility criteria** | 6 | Describe the rationale for including particular study designs related to equity research questions. | 4 - 5 |
|  | 6A | Describe the rationale for including the outcomes - e.g. how these are relevant to reducing inequity. | 4 - 5 |
| **Information sources** | 7 | Describe information sources (e.g. health, non-health, and grey literature sources) that were searched that are of specific relevance to address the equity questions of the review. | 4 |
| **Search** | 8 | Describe the broad search strategy and terms used to address equity questions of the review. | 4 & S4 File |
| **Data items** | 11 | List and define data items related to equity, where such data were sought (e.g. using PROGRESS-Plus or other criteria, context). | 5 & S5 File |
| **Synthesis of results** | 14 | Describe methods of synthesizing findings on health inequities (e.g. presenting both relative and absolute differences between groups). | 6 |
| **Additional analyses** | 16 | Describe methods of additional synthesis approaches related to equity questions, if done, indicating which were pre-specified | 6 |
| **Results** |  |  |  |
| **Study characteristics** | 18 | Present the population characteristics that relate to the equity questions across the relevant PROGRESS-Plus or other factors of interest. | 8-13 & 17-18 |
| **Synthesis of results** | 21 | Present the results of synthesizing findings on inequities (see 14). | 17-18 & S8 File |
| **Additional analysis** | 23 | Give the results of additional synthesis approaches related to equity objectives, if done, (see 16). | 17-18 & S8 File |
| **Discussion** |  |  |  |
| **Conclusions** | 26 | Present extent and limits of applicability to disadvantaged populations of interest and describe the evidence and logic underlying those judgments. | 18-19 |
|  | 26A | Provide implications for research, practice or policy related to equity where relevant (e.g. types of research needed to address unanswered questions). | 19-20 |
