# Supplementary material for: Mobile interventions targeting common mental disorders among pregnant and postpartum women: An equity-focused systematic review
Source: PLoS One. 2021 Oct 29;16(10):e0259474. doi: 10.1371/journal.pone.0259474 (PMC8555821; doi:10.1371/journal.pone.0259474)
Supplement: S3 File — (DOCX) [file pone.0259474.s003.docx]

**Mobile interventions targeting common mental disorders among pregnant and postpartum women: An equity-focused systematic review**

**Appendix III: Overview of our collaborative research approach**

Project partners were invited through our networks and have ensured their commitment to meaningfully support our work, now and in the post COVID-19 era. They have been engaged heterogeneously across the process and have been categorized into four major groups: researchers; practitioners and service providers; patients and consumers of care; and patient organizations. Table S1 represents our collaborative research matrix which has been developed using the stakeholder engagement guidance put forward by Conacannon and colleagues (Concannon 2019). The matrix describes project partner groups and qualifies their roles, contributions, and level of engagement, as well as the time frame and mode of such engagement.

**Table S1.** Collaborative Research Matrix

| **Stage/**  **Time Frame** | **Research activity** | **Project partner groups** | | | | | |
| --- | --- | --- | --- | --- | --- | --- | --- |
|  |  | Researchers | | Practitioners/ providers | | Patients/ consumers | Patient organizations |
|  |  | Health services and equity methods researchers | Research organisations | Psychiatrists | Psychologists | Pregnant and postpartum women | Patient advocacy group |
| Project planning phase before the formulation of project scope and research questions | Building research capacity | **R**: Decision maker  **M**: virtual meetings, emails, phone calls | **R**: Advisors  **M**: emails | **R**: Decision maker  **M**: virtual meetings, emails, phone calls | **R**: Decision maker  **M**: virtual meetings, emails, phone calls | N | N |
|  | Training to work with stakeholders | **R**: Decision maker  **M**: virtual meetings, emails, phone calls | N | **R**: Decision maker  **M**: virtual meetings, emails, phone calls | N | N | N |
|  | Prioritizing evidence gaps | **R**: Decision maker  **M**: virtual meetings, emails, phone calls | **R**: Advisors  **M**: emails | **R**: Decision maker  **M**: virtual meetings, emails, phone calls | **R**: Decision maker  **M**: virtual meetings, emails, phone calls | N | N |
|  | Choosing research topics | **R**: Decision maker  **M**: virtual meetings, emails, phone calls | **R:** Feedback  **M**: emails | **R**: Decision maker  **M**: virtual meetings, emails, phone calls | **R**: Decision maker  **M**: virtual meetings, emails, phone calls | N | N |
| Project planning phase after the formulation of project scope and research questions | Defining scope and research questions | **R**: Decision maker  **M**: virtual meetings, emails, phone calls | **R**: Feedback  **M**: emails | **R**: Decision maker  **M**: virtual meetings, emails, phone calls | **R**: Decision maker  **M**: emails, Google documents | N | N |
|  | Choosing relevant outcomes | **R**: Decision maker  **M**: virtual meetings, emails, phone calls | N | **R**: Decision maker  **M**: virtual meetings, emails, phone calls | **R**: Advisor  **M**: virtual meetings, emails, phone calls | N | N |
|  | Designing research protocol | **R**: Decision maker  **M**: virtual meetings, emails, phone calls | N | **R**: Decision maker  **M**: virtual meetings, emails, phone calls | N | N | N |
| Project execution phase. It included performing all research activities that have been proposed in the protocol and the writing of this work | Performing research activities (as described in methods) | **R**: Decision maker  **M**: virtual meetings, emails, Google documents | N | **R**: Decision maker  **M**: virtual meetings, emails, Google documents | **R**: Decision maker  **M**: virtual meetings, emails, Google documents | N | N |
|  | Identifying findings | **R**: Decision maker  **M**: virtual meetings, emails, Google documents | N | **R**: Decision maker  **M**: virtual meetings, emails, Google documents | **R**: Decision maker  **M**: virtual meetings, emails, Google documents | **R**: Advisor  **M**: virtual meetings, phone calls, emails and messages | **R**: Decision makers  **M**: virtual meetings, phone calls, emails and messages |
|  | Interpreting findings | **R**: Decision maker  **M**: virtual meetings, emails, Google documents | N | **R**: Decision maker  **M**: virtual meetings, emails, Google documents | **R**: Decision maker  **M**: virtual meetings, emails, Google documents | **R**: Decision makers  **M**: virtual meetings, phone calls, emails and messages | **R**: Decision makers  **M**: virtual meetings, phone calls, emails and messages |
|  | Identifying implications for future research | **R**: Decision maker  **M**: virtual meetings, emails, Google documents | N | **R**: Decision maker  **M**: virtual meetings, emails, Google documents | **R**: Decision maker  **M**: virtual meetings, emails, Google documents | **R**: Decision makers  **M**: virtual meetings, phone calls, emails and messages | **R**: Decision maker  **M**: virtual meetings, emails, Google documents |
| end-of-project phase and thereafter. It includes all end-of-project KT activities and their evaluation | Designing End-of- Project KT | **R**: Decision maker  **M**: virtual meetings, emails, Google documents | **R:** Advisor  **M:** emails and phone calls | **R**: Decision maker  **M**: virtual meetings, emails, Google documents | **R**: Decision maker  **M**: virtual meetings, emails, Google documents | **R**: Decision maker  **M**: virtual meetings, emails, Google documents | **R**: Decision maker  **M**: virtual meetings, emails, Google documents |
|  | Disseminating findings | **R**: Decision maker  **M**: virtual meetings, emails, Google documents | **R**: Decision maker  **M**: virtual meetings, emails, Google documents | **R**: Decision maker  **M**: virtual meetings, emails, Google documents | **R**: Decision maker  **M**: virtual meetings, emails, Google documents | **R**: Decision maker  **M**: virtual meetings, emails, Google documents | **R**: Decision maker  **M**: virtual meetings, emails, Google documents |
|  | Evaluating KT | **R**: Decision maker  **M**: virtual meetings, emails, Google documents | **R**: Advisors  **M**: virtual meetings, emails, Google documents | **R**: Decision maker  **M**: virtual meetings, emails, Google documents | **R**: Decision maker  **M**: virtual meetings, emails, Google documents | **R**: Advisors  **M**: virtual meetings, emails, Google documents | **R**: Decision maker  **M**: virtual meetings, emails, Google documents |
|  | Evaluating engagement | **R**: Decision maker  **M**: virtual meetings, emails, Google documents | **R**: Decision maker  **M**: virtual meetings, emails, Google documents | **R**: Decision maker  **M**: virtual meetings, emails, Google documents | **R**: Decision maker  **M**: virtual meetings, emails, Google documents | **R**: Decision maker  **M**: virtual meetings, emails, Google documents | **R**: Decision maker  **M**: virtual meetings, emails, Google documents |

Moreover, we have consulted with multiple experts in the field of knowledge translation to inform the design and execution of our KT plan. Our team included a scientist representative (OM), as well as communication experts from our partner organizations with working experience in KT. Our experts have, and continue to, consult us on the conceptualization of the knowledge translation plan, design of knowledge diffusion and dissemination interventions, and evaluation of our knowledge translation impact. We have also reached an agreement with the Bruyère Research Institute to disseminate our knowledge through their communication networks (e.g., mailbag, Life Changing Day, research grand rounds), and highlight our publications through their social media channels.

**Researchers**: Collaborating with scientists with expertise in health service and policy and equity methods has informed our decision to apply an equity lens to our work and guided the process to properly do so. The Bruyère Research Institute, a research-focused organization, has supported this project, both financially and logistically, and provided the means to communicate our early findings to different stakeholders through utilizing their internal communication network, or by supporting our external endeavours to engage key stakeholders groups. Further, the University of Ottawa, Faculty of Medicine has provided the venue to present our methods (i.e., protocol) and early equity findings (Please see chapter 1.3) to a group of scientists and peer investigators, who provided feedback on our work and refined the process by which we report our results.

**Practitioners and service providers**: Choosing mobile interventions to prevent and manage common mental disorders among pregnant and postpartum women is a shared health decision that requires buy-in from not only patients, but also practitioners and providers of care. We have, therefore, engaged multiple representatives of practitioners and care providers, such as psychiatrists, psychologists, Maternal psychotherapists, maternal health providers, as well as graduate and undergraduate medical students. Our partners have guided the process of conceptualizing methods, selecting clinically meaningful outcomes, and interpreting findings in the context of practice and care delivery. As well, they have collaborated on the design of our end-of-project KT plan.

**Patients**: We have partnered with “patient representatives”, defined as pregnant and postpartum women with lived experience of stress and common mental disorders, and have worked with them to attune our work to their needs and values. Our patient representatives chose to remain anonymous for privacy concerns. We have worked with our patient representatives to interpret our data, decide on clinically meaningful/ important findings, identify gaps in knowledge, and plan for end-of-project knowledge translation.

**Patient organizations**: “FemTech” or “Female Technology” represents an emerging sector that focuses on using technology-based care to improve the health and wellbeing of women. We have partnered with “FemTech Focus”, a patient-oriented organization well-rooted among women opinion and community leaders with a global reach to thousands of women. We have consulted experts from “FemTech Focus” to help us interpret our data, tailor our findings to the context of pregnant and postpartum women and move towards an effective end-of-project knowledge translation. Our deliberations have highlighted the need to advocate for the democratization of women’s health and call for supporting women to take control of their own mental health during and after pregnancy.

**References**

Concannon TW, Grant S, Welch V, Petkovic J, Selby J, Crowe S, Synnot A, Greer-Smith R, Mayo-Wilson E, Tambor E, Tugwell P. Practical guidance for involving stakeholders in health research. Journal of general internal medicine. 2019 Mar;34(3):458-63.
