# Supplementary material for: Mobile interventions targeting common mental disorders among pregnant and postpartum women: An equity-focused systematic review
Source: PLoS One. 2021 Oct 29;16(10):e0259474. doi: 10.1371/journal.pone.0259474 (PMC8555821; doi:10.1371/journal.pone.0259474)
Supplement: S4 File — (DOCX) [file pone.0259474.s004.docx]

**Mobile interventions targeting common mental disorders among pregnant and postpartum women: An equity-focused systematic review**

**Appendix IV: Search strategy and grey literature outputs**

- **Primary search strategy of MEDLINE via OVID**

Date of search: June 26, 2020; Date of updated search: Jan 4, 2021

| **MEDLINE via OVID** |
| --- |
| exp pregnancy/ or pregnant women/ |
| exp prenatal care/ or exp perinatal care/ or exp postnatal care/ or exp postpartum period/ |
| (pregnan* or prenatal or pre-natal or perinatal or peri-natal or postnatal or post-natal or postpartum or post-partum or ((perinatal or prenatal or postpartum or postnatal) adj2 care*) or ((peri-natal or pre-natal or post-partum or post-natal) adj2 care*)).ti,ab,kf. |
| 1 or 2 or 3 |
| mental health/ or exp mental disorders/ or depression/ or anxiety/ or stress disorder, post-traumatic/ or stress, psychological/ or psychological distress/ |
| (mental health* or wellbeing or well-being or (mental adj2 health*) or psychology or psychiatry or psych* or stress* or depress* or anxiet* or anxi* or anxious* or posttrauma* or post-trauma* or trauma or PTSD).ti,ab,kf. |
| 5 or 6 |
| smartphone/ or exp cell phones/ or exp computers, handheld/ or mobile application/ or wireless technology/ or text messaging/ |
| (mhealth* or m-health*).ti,ab,kf. |
| (((mobile or cell* or portable) adj2 phone*) or cellphone* or cell-phone* or smartphone* or smart-phone*).ti,ab,kf. |
| ((mobile adj2 application*) or (phone adj2 application*) or app or apps).ti,ab,kf. |
| ((mobile adj2 health) or (mobile adj2 tech*)).ti,ab,kf. |
| (text* or sms or (text* adj2 messag*) or (sms adj2 messag*)).ti,ab,kf. |
| 8 or 9 or 10 or 11 or 12 or 13 |
| 4 and 7 and 14 |

- **Grey literature search strategy and outputs**

We have developed a focused grey literature search strategy to capture reports of experiments that meet our eligibility criteria but have not been published or indexed in any of the primary search sources proposed in the review protocol.

We first started by searching *OpenGrey* for grey literature originating from Europe, using a combination of truncated keywords presented in table 1.

We then used a [custom google search engine](https://libguides.nus.edu.sg/c.php?g=145495&p=954401) (developed by the National University of Singapore Library) to search the websites of over 1500 Non-governmental organizations ([found here](https://docs.google.com/spreadsheets/d/1ifZrdxJay_bIQtb1V6OAJBxMAEIJV8vzrvN3zzXD7jM/edit?usp=sharing)) and over 400 Intergovernmental organizations ([found here](https://docs.google.com/spreadsheets/d/1aQj-MOSep_2S5yja_IxDRpRwqdhMXzxzTmtDZO4UK40/edit?usp=sharing)). We set a search limit sensitive to the relevance of results; we explored publications from websites of NGOs and IGOs in the order that was yielded by the search, but tested the sensitivity of these results (i.e how relevant the organizations and publications were to our review scope) beyond the 20-website level. If relevant websites with publications existed beyond the 20-website limit, we continued the search process for another 5 websites and performed the sensitivity testing again and until search results were deemed completely irrelevant. Search results are presented in tables 2 and 3.

Finally, we searched for pregnancy-specific organizations and associations that provided care, services, or representation to pregnant or postpartum women at the national and international level using a focused Google search strategy and excluding ad-forwarded websites. We used a similar search limit sensitive to whether these organizations and any publications found on their websites were relevant to our review scope at the 20-website level. If relevant websites with publications existed beyond the 20-website limit, we continued the search process for another 5 websites and performed the sensitivity testing again and until search results were deemed completely irrelevant. Search results are presented in table 4.

Grey literature search results:

**Table 1: Search strategy for OpenGrey**

| Terms used | Records captured | Records screened |
| --- | --- | --- |
| pregnan* AND psych* | 232 | 232 |
| post-partum AND psych* | 70 | 70 |
| postpartum AND psych* | 41 | 41 |
| postnatal AND psych* | 87 | 87 |
| pregnan* AND mobile | 4 | 4 |
| post-partum AND mobile | 4 | 4 |
| postpartum AND mobile | 0 | 0 |
| postnatal AND mobile | 0 | 0 |
| pregnan* AND mental | 22 | 22 |
| post-partum AND mental | 2 | 2 |
| postpartum AND mental | 1 | 1 |
| postnatal AND mental | 15 | 15 |
| pregnan* AND digital | 6 | 6 |
| postpartum AND digital | 0 | 0 |
| post-partum AND digital | 0 | 0 |
| postnatal AND digital | 0 | 0 |
| TOTAL | 484 | 484 |

**Table 2: Non-governmental websites search results**

| **Website** | **Publications relevant to our review scope?** |
| --- | --- |
| WHO Maternal Health  <https://www.who.int/data/maternal-newborn-child-adolescent-ageing> | No |
| International Atomic Energy Agency  <https://www.iaea.org/resources/rpop/health-professionals/radiology/pregnant-women> | No |
| Publication: The Importance of a Life Course Approach to Health: Chronic Disease Risk from Preconception through Adolescence and Adulthood  <https://www.who.int/life-course/publications/life-course-approach-to-health.pdf> | No |
| mHealth New horizons for health through mobile technologies  <https://www.who.int/goe/publications/goe_mhealth_web.pdf> | No |
| Evidence of accessing antenatal care information via social media platforms supports mental wellbeing in COVID-19 epidemic  <https://www.who.int/bulletin/online_first/20-255489.pdf> | No |
| United Nations: Crimes against humanity  <https://www.un.org/en/genocideprevention/crimes-against-humanity.shtml> | No |
| Africa Wired: Portable ultrasound device to tackle child mortality  <https://www.un.org/africarenewal/magazine/december-2016-march-2017/africa-wired-portable-ultrasound-device-tackle-child-mortality> | No |
| Systematic review on the health effects of exposure to radiofrequency electromagnetic fields from mobile phone base stations  <https://www.who.int/bulletin/volumes/88/12/09-071852/en/> | No |
| United Nations: War crimes  <https://www.un.org/en/genocideprevention/war-crimes.shtml> | No |
| WHO: Urban green spaces and health  <https://www.euro.who.int/__data/assets/pdf_file/0005/321971/Urban-green-spaces-and-health-review-evidence.pdf?ua=1> | No |
| One stillbirth occurs every 16 seconds, according to first ever joint UN estimates  <https://www.who.int/news/item/08-10-2020-one-stillbirth-occurs-every-16-seconds-according-to-first-ever-joint-un-estimates> | No |
| WHO: Telemedicine: Opportunities and developments in member states  <https://www.who.int/goe/publications/goe_telemedicine_2010.pdf> | No |
| United Nations: Policy Brief: The Impact of COVID-19 on Women  <https://www.un.org/sexualviolenceinconflict/wp-content/uploads/2020/06/report/policy-brief-the-impact-of-covid-19-on-women/policy-brief-the-impact-of-covid-19-on-women-en-1.pdf> | No |
| UNESCO: Digital inclusion for low-skilled and low-literate people: a landscape review  <https://unesdoc.unesco.org/ark:/48223/pf0000261791> | No |
| UNESCO: Mapping of online articles on Covid-19 and Gender  <https://en.unesco.org/news/mapping-online-articles-covid-19-and-gender> | No |
| WHO: Mental health promotion and mental health care in refugees and migrants Technical guidance  <https://www.euro.who.int/__data/assets/pdf_file/0004/386563/mental-health-eng.pdf?ua=1> | No |
| United Nations: Realizing the Sustainable Development Goals by, for and with persons with disabilities  <https://www.un.org/development/desa/disabilities/wp-content/uploads/sites/15/2019/07/disability-report-chapter2.pdf> | No |
| UNESCO strategy on education for health and well-being: contributing to the Sustainable Development Goals  <https://unesdoc.unesco.org/ark:/48223/pf0000246453> | No |
| WHO: Child marriages: 39 000 every day  <https://www.who.int/mediacentre/news/releases/2013/child_marriage_20130307/en/> | No |
| UNHCR: Human Rights Standards and Practice for the Police  <https://www.ohchr.org/documents/publications/training5add3en.pdf> | No |
| **Results were deemed irrelevant for the scope of our review at the 20-website level. Search terminated.** | |

**Table 3: Intergovernmental organizations websites search results**

| **Website** | **Publications relevant to our review scope?** |
| --- | --- |
| mHealth Alliance: mHealth and MNCH: State of the Evidence  <https://www.gfmer.ch/mhealth/coursefiles2013/mhealthmnch-evidence-final.pdf> | No |
| Geneva Foundation for Medical Education and Research: Sexual and Reproductive mHealth Better Access to Health Care through Mobile phones  <https://www.gfmer.ch/mhealth/pdf/Sexual-Reproductive-mHealth-Nurmi-2013.pdf> | No |
| OCHA: Adolescent Girls Fact Sheet  <https://m.reliefweb.int/report/1294976/world/adolescent-girls-fact-sheet?lang=ru> | No |
| World Bank: Gender Dimensions of the COVID-19 Pandemic  <https://openknowledge.worldbank.org/bitstream/handle/10986/33622/Gender-Dimensions-of-the-COVID-19-Pandemic.pdf> | No |
| United Nations: Policy Brief: The Impact of COVID-19 on Women  <https://reliefweb.int/sites/reliefweb.int/files/resources/policy-brief-the-impact-of-covid-19-on-women-en.pdf> | No |
| USAID: Delivering sexual and reproductive health services to young people  <https://www.msichoices.org/media/2117/delivering-sexual-and-reproductive-health-services-to-young-people.pdf> | No |
| OCHA: South Asia: Reproductive health care being restored in Tsunami-hit areas  <https://reliefweb.int/report/indonesia/south-asia-reproductive-health-care-being-restored-tsunami-hit-areas> | No |
| The PATH Organization: Life Planning Skills: A curriculum for young people in africa Uganda version  <https://path.azureedge.net/media/documents/HIV-TB_aya_lps_facilitator_ugan_sec3.pdf> | No |
| Kenya Human Rights Commission:  Teenage Pregnancy and Unsafe Abortion The Case of Korogocho Slums  <https://www.khrc.or.ke/mobile-publications/equality-and-anti-discrimination/69-teenage-pregnancy-and-abortion-case-study/file.html> | No |
| IPPF Western Hemisphere region: Guidelines on Sexual and  Reproductive Healthcare for Youth  <https://www.ippfwhr.org/wp-content/uploads/2018/08/Youth_Meeting_-_English_-_NOV__1_.pdf> | No |
| World Bank: Disease Control Priorities, Third Edition (Volume 4)  <https://openknowledge.worldbank.org/bitstream/handle/10986/23832/9781464804267.pdf;sequence=3> | No |
| Food Research and Action Centre: Making WIC Work Better: Strategies to Reach More Women and Children and Strengthen Benefits Use  <https://frac.org/wp-content/uploads/Making-WIC-Work-Better-Full-Report.pdf> | No |
| Population council: Unintended Pregnancy and Abortion in India:  Country Profile Report  <https://www.popcouncil.org/uploads/pdfs/2014STEPUP_IndiaCountryProfile.pdf> | No |
| Human Rights Watch: Barriers to HIV Services and Treatment for Persons  with Disabilities in Zambia  <https://www.hrw.org/reports/zambia0714_ForUpload_1.pdf> | No |
| Evidence Consortium on Women’s Group: The Impact of COVID-19 on Opportunities for Adolescent Girls and the Role of Girls’ Groups  <https://www.popcouncil.org/uploads/pdfs/2020PGY_ECWG-AdolescentGirlsCOVID.pdf> | No |
| IDRC: Feminist advocacy, family lawand violence against women  <https://www.idrc.ca/sites/default/files/sp/Images/IDRC%20Books/idl-57272.pdf> | No |
| Food Research and Action Centre: Addressing Food Insecurity:  A Toolkit for Pediatricians  <https://frac.org/wp-content/uploads/frac-aap-toolkit.pdf> | No |
| OCHA: South Asia: Tsunami & Health Situation Report # 34  <https://m.reliefweb.int/report/411552?lang=ru> | No |
| The 10th International Congress on Adolescent Health: Theme: Bridging clinical and public health perspectives to promote adolescent health  <https://iaah.org/wp-content/uploads/2019/05/Posters-Presentations-Symposiums-2013-World-Congress.pdf> | No |
| The 31st Conference of the International Society for Environmental Epidemiology: Program and abstracts  <https://www.iseepi.org/common/Uploaded%20files/PROGRAM-ISEE-A4_FINAL.pdf> | No |
| **Results were deemed irrelevant for the scope of our review at the 20-website level. Search terminated.** | |

**Table 4: National and international pregnancy-specific organizations and associations**

| **Website** | **Publications relevant to our review scope?** |
| --- | --- |
| Pregnancy Care Canada  [www.pregnancycarecanada.ca](http://www.pregnancycarecanada.ca) | No |
| American Pregnancy Association  [www.americanpregnancy.org](http://www.americanpregnancy.org) | No |
| Pregnancy Centres Network UK  <https://pregnancycentresnetwork.org.uk/> | No |
| The MoTHERS Program  [www.themothersprogram.ca](http://www.themothersprogram.ca) | No |
| Pregnancy Info  [www.pregnancyinfo.ca](http://www.pregnancyinfo.ca) | No |
| Women’s Health: Pregnancy  [www.womenshealth.gov/pregnancy](http://www.womenshealth.gov/pregnancy) | No |
| Mother to Baby  [www.mothertobaby.org](http://www.mothertobaby.org) | No |
| Postpartum Support International  [www.postpartum.net](http://www.postpartum.net) | No |
| Birth Companions  <https://www.birthcompanions.org.uk/> | No |
| Beginnings Family Services  [www.beginnings.ca/](http://www.beginnings.ca/) | No |
| Pregnancy help  [www.pregnancyhelp.ca](http://www.pregnancyhelp.ca) | No |
| St. Stephen Community House  [www.sschto.ca/adults/pregnant-women](http://www.sschto.ca/adults/pregnant-women) | No |
| Guttmacher Institute: United States  Pregnancy  <https://www.guttmacher.org/united-states/pregnancy> | No |
| Ontario Settlement: Pregnancy and Health  <https://settlement.org/ontario/health/sexual-and-reproductive-health/pregnancy-and-birth/i-am-pregnant-where-can-i-find-services/> | No |
| The British Columbia Association of Pregnancy Outreach Programs  <https://www.bcapop.ca/> | No |
| Perinatal Services of British Columbia  <http://www.perinatalservicesbc.ca/> | No |
| Crisis Pregnancy Centre of New York  <https://www.cpcny.org/> | No |
| Anxiety Canada: Moms to Be  <https://www.anxietycanada.com/general/moms-to-be/> | No |
| Pacific Postpartum Support Society  <http://postpartum.org/> | No |
| Maternity Action UK  <https://maternityaction.org.uk/what-we-do/our-research/> | No |
| **Results were deemed irrelevant for the scope of our review at the 20-website level. Search terminated.** | |
