# Supplementary material for: Mobile interventions targeting common mental disorders among pregnant and postpartum women: An equity-focused systematic review
Source: PLoS One. 2021 Oct 29;16(10):e0259474. doi: 10.1371/journal.pone.0259474 (PMC8555821; doi:10.1371/journal.pone.0259474)
Supplement: S5 File — (DOCX) [file pone.0259474.s005.docx]

**Mobile interventions targeting common mental disorders among pregnant and postpartum women: An equity-focused systematic review**

**Appendix V: Information extracted from primary studies using the standardised data extraction form**

| Study ID | Study citation | | |
| --- | --- | --- | --- |
|  |  | | |
| Study design | Specific study design structure | | |
|  |  | | |
| Background, rationale, and context  (Any epidemiological, statistical, contextual, or equity-related information used by investigators to introduce the topic) (**E)** | | | Study objectives and aims as described by the investigators |
|  | | |  |
| Study location (city, country) | Study setting (in which participants were recruited or the intervention delivered) | | |
|  |  | | |
| Target population as described by investigators | Pregnancy period (antenatal, perinatal, postnatal) | Pregnancy age (in weeks or trimesters) | Is the population being selected based on a PROGRESS+ criteria? (**E)** |
|  |  |  |  |
| Eligibility criteria | Recruitment procedures | Recruitment period | Sample size at allocation  (Total and per study arm) |
|  |  |  |  |
| Name of the intervention | Focus/ purpose of the intervention | Is the intervention using mobile technology as the primary method of delivery?  (Yes/ No) | |
|  |  |  | |
| Nature of the intervention  (Smartphone application, text messaging program, combination, other) | | Design of the intervention  (information, knowledge, therapy technique, or support provided) | Delivery of the intervention  (length, dosage, and by whom) |
|  | |  |  |
| Description of the comparison/ control treatment | | | |
|  | | | |
| Time of data collection | Nature of baseline data collection | Nature of follow-up data collection  (Face-to-face interviews, online questionnaires, etc.) | |
|  |  |  | |
| Demographic data analysis procedures | | Outcome measurement data analysis procedures | |
|  | |  | |
| Were the results analyzed/stratified based on a PROGRESS+ criteria? (**E)** | | | |
|  | | | |
| Significance level set by investigators | | Analysis principle  (Intent-to-treat, per-protocol, unidentified) | |
|  | |  | |
| Sample size at follow up  (Total and per study arm) | Baseline demographics as described by investigators | Equity-related (PROGRESS+) baseline demographics (**E)** | |
|  |  |  | |
| Outcome domain #1 | Outcome measurement tool #1 | Summary of result #1 | |
|  |  |  | |
| Outcome domain #2 | Outcome measurement tool #2 | Summary of result #2 | |
|  |  |  | |
| Outcome domain #3 | Outcome measurement tool #3 | Summary of result #3 | |
|  |  |  | |
| Outcome domain #4 | Outcome measurement tool #4 | Summary of result #4 | |
|  |  |  | |
| Equity-related findings as described by investigators **(E)** | | | |
|  | | | |
| Study conclusions | Study limitations | Funding | Conflicts of interest |
|  |  |  |  |

**(E) Equity was considered when extracting data at this level**
