# Supplementary material for: Mobile interventions targeting common mental disorders among pregnant and postpartum women: An equity-focused systematic review
Source: PLoS One. 2021 Oct 29;16(10):e0259474. doi: 10.1371/journal.pone.0259474 (PMC8555821; doi:10.1371/journal.pone.0259474)
Supplement: S6 File — (DOCX) [file pone.0259474.s006.docx]

**Mobile interventions targeting common mental disorders among pregnant and postpartum women: An equity-focused systematic review**

**Appendix VI: Critical appraisal visuals (by outcome)**

**1. Visual representations of critically appraising results on the severity of depression symptoms**


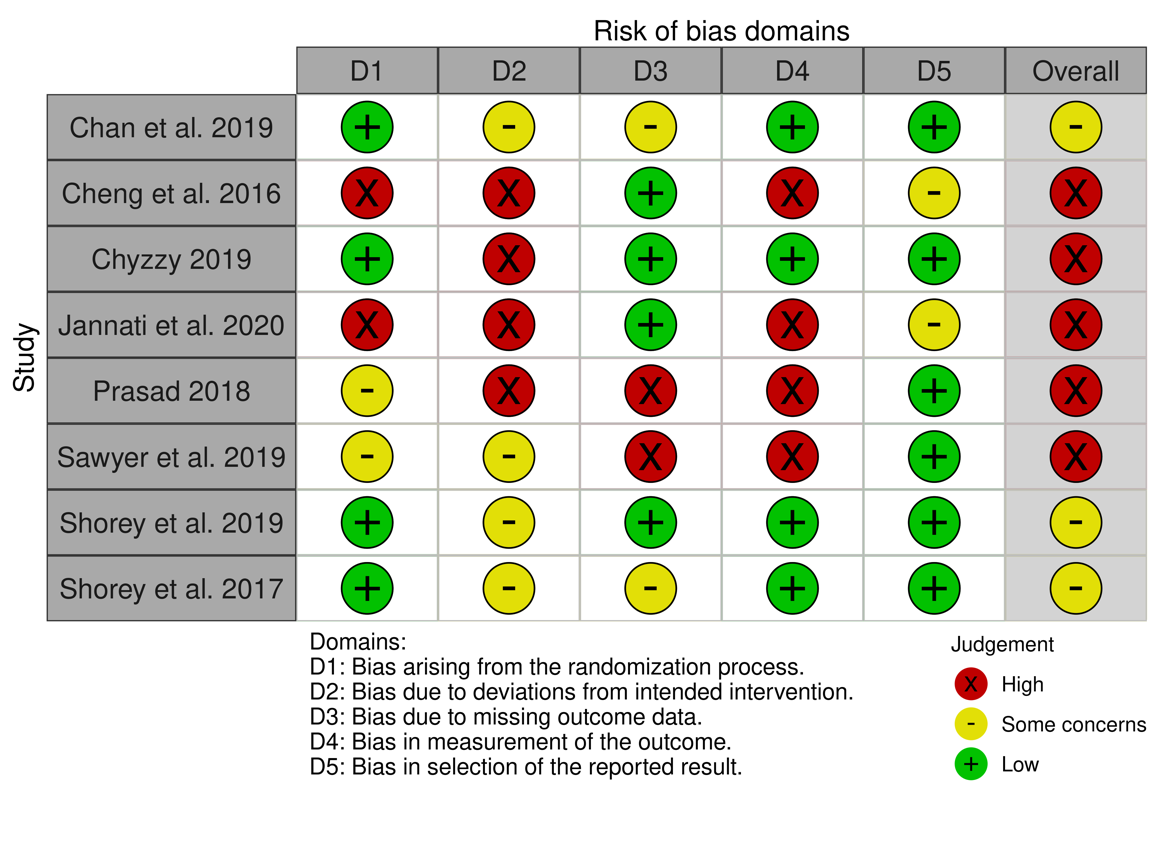


**Fig 1. Risk of bias assessments on the severity of depression symptoms from randomized controlled trials of interventions using the Cochrane *Risk of Bias 2.0* tool (ROB 2.0) - study level**


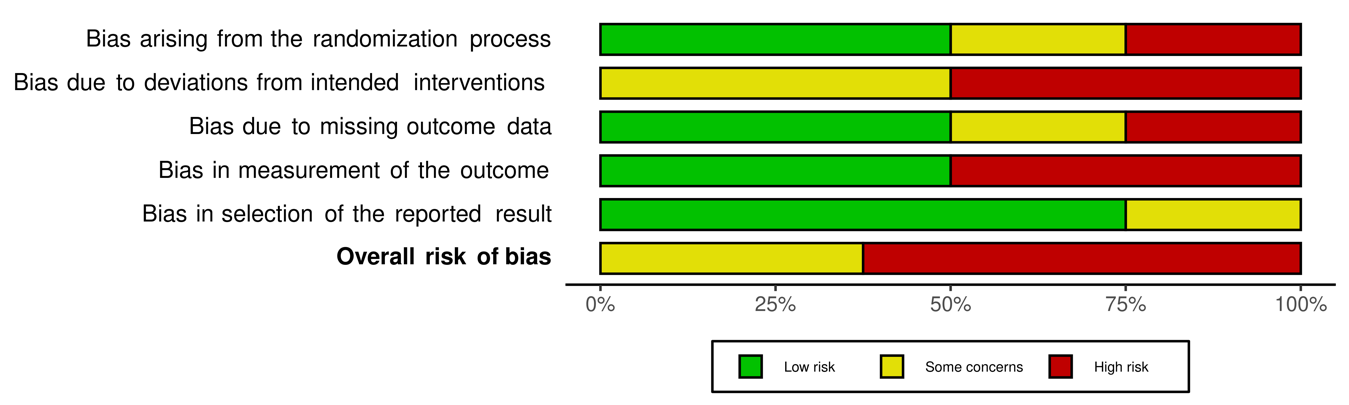


**Fig 2. Risk of bias assessments on the severity of depression symptoms from randomized controlled trials of interventions using the Cochrane *Risk of Bias 2.0* tool (ROB 2.0) - across studies**


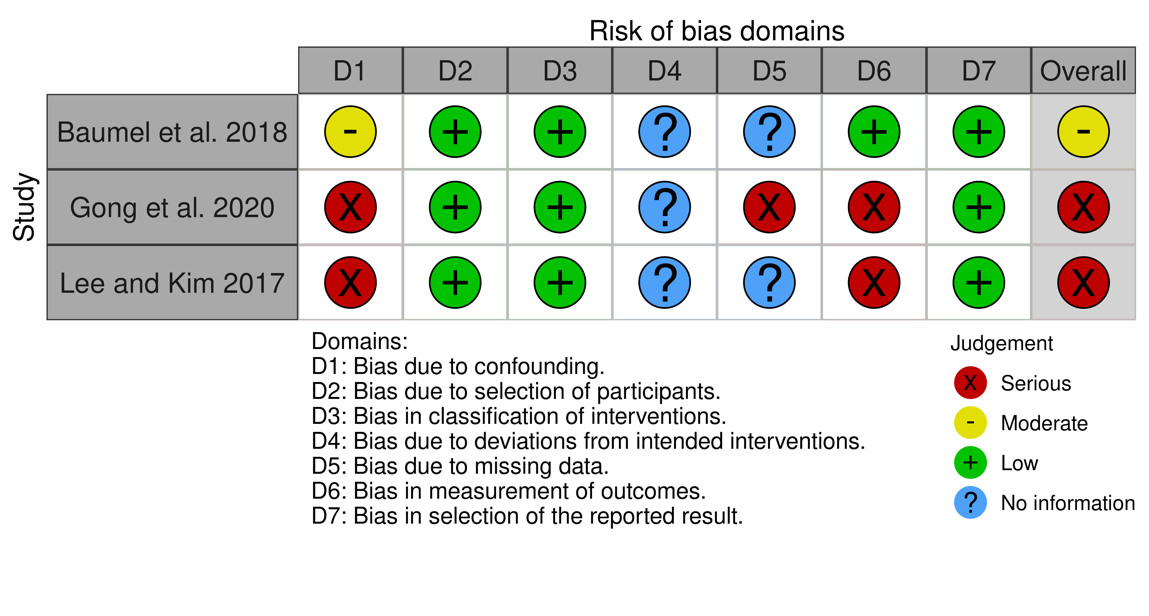


**Fig 3. Risk of bias assessments on the severity of depression symptoms from non-randomized controlled studies using the Cochrane *Risk of Bias In Non-randomized Studies of Interventions* Tool (ROBINS-I) - study level**


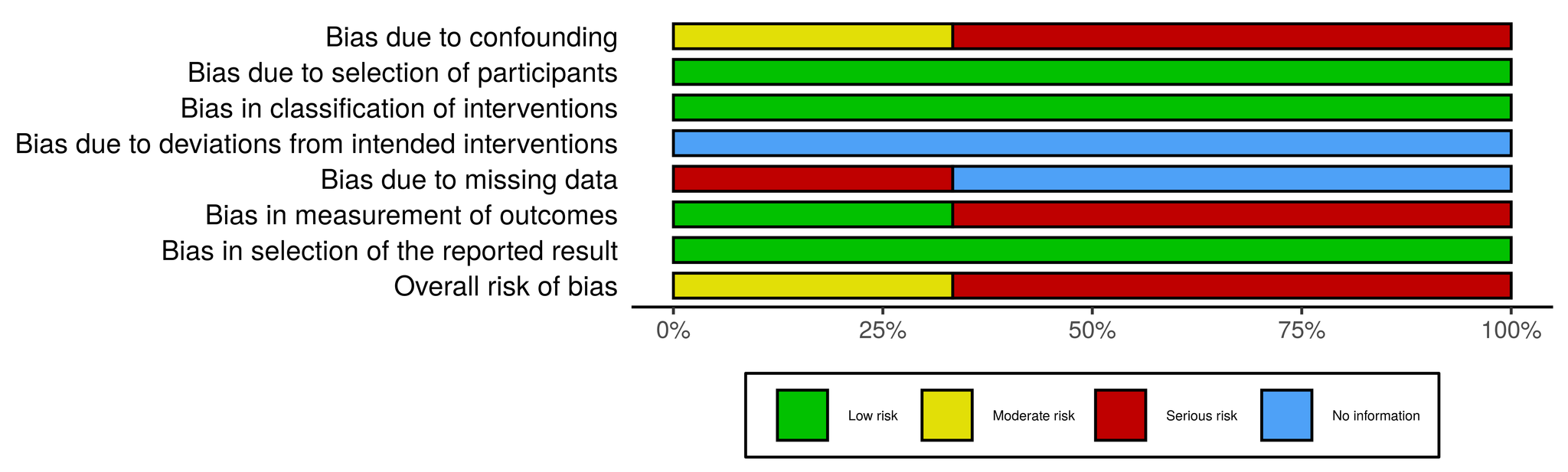


**Fig 4. Risk of bias assessments on the severity of depression symptoms from non-randomized controlled studies using the Cochrane *Risk of Bias In Non-randomized Studies of Interventions* Tool (ROBINS-I) - across studies**

**2. Visual representations of critically appraising results on the severity of anxiety symptoms**


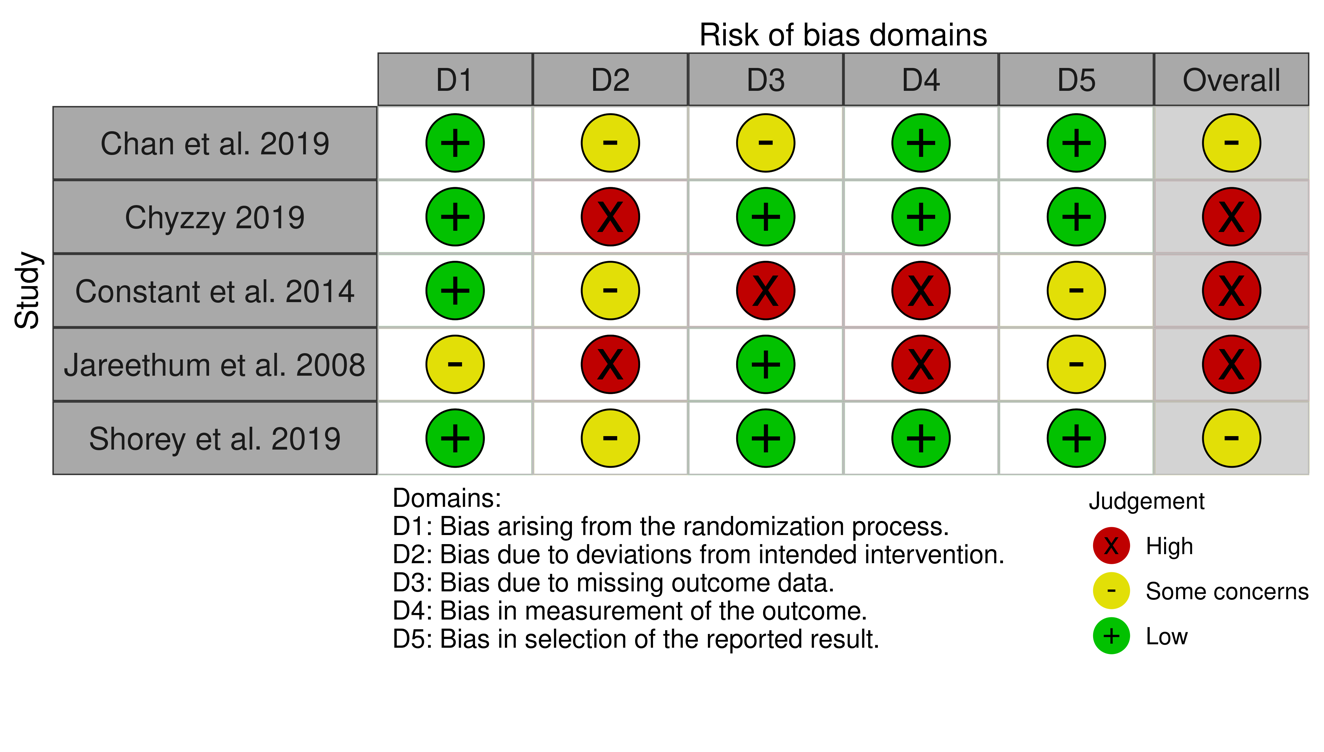


**Fig 5. Risk of bias assessments on the severity of anxiety symptoms from randomized controlled trials of interventions using the Cochrane *Risk of Bias 2.0* tool (ROB 2.0) - study level**


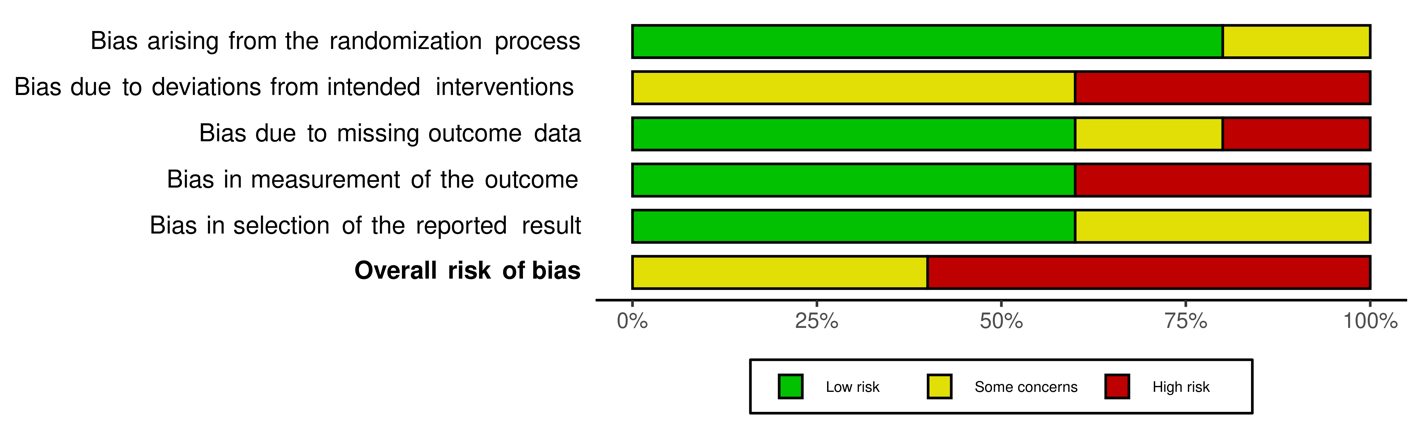


**Fig 6. Risk of bias assessments on the severity of anxiety symptoms from randomized controlled trials of interventions using the Cochrane *Risk of Bias 2.0* tool (ROB 2.0) - across studies**

**Note**: No results from non-randomized controlled studies were found on the severity of anxiety symptoms

**3. Visual representations of critically appraising results on the psychological wellbeing and distress**


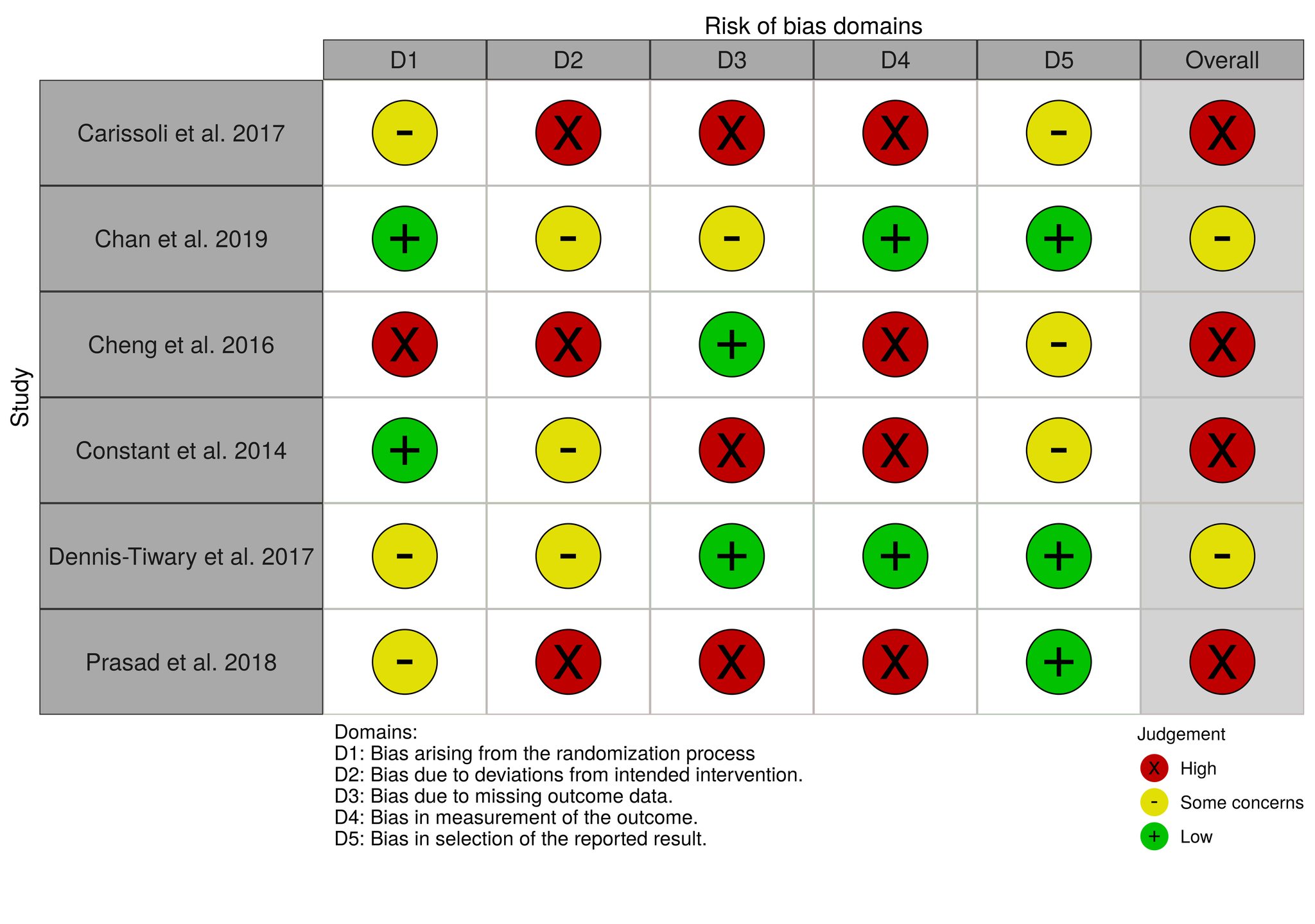


**Figure 7**: Risk of bias assessments on psychological stress from randomized controlled trials of interventions using the Cochrane *Risk of Bias 2.0* tool (ROB 2.0) - study level


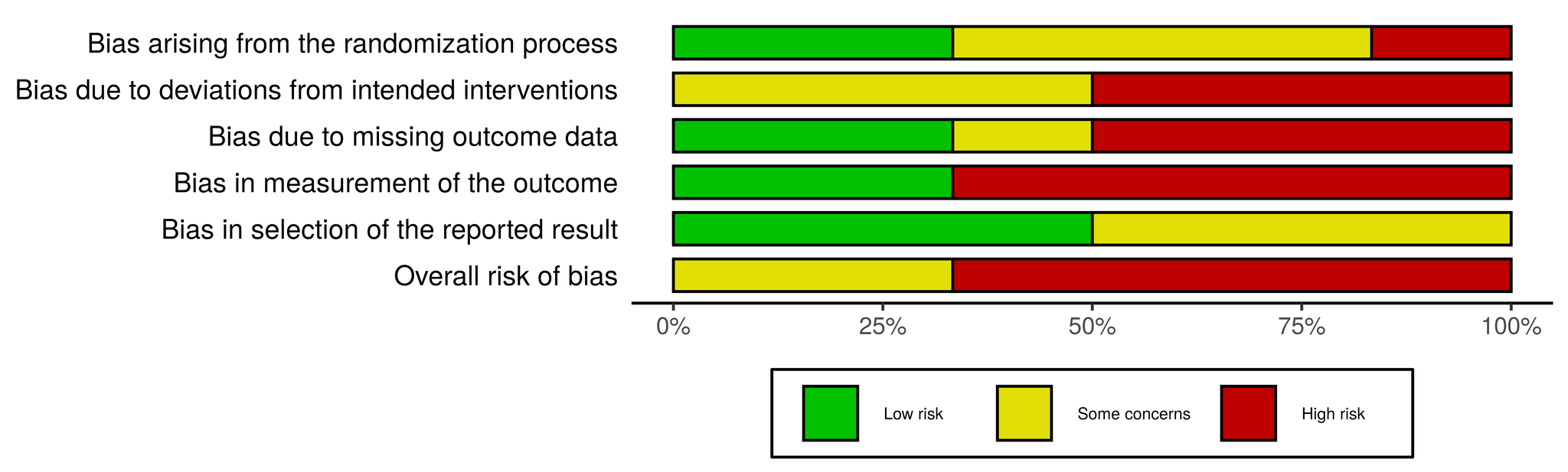


**Figure 8**: Risk of bias assessments on psychological stress from randomized controlled trials of interventions using the Cochrane *Risk of Bias 2.0* tool (ROB 2.0) - across studies


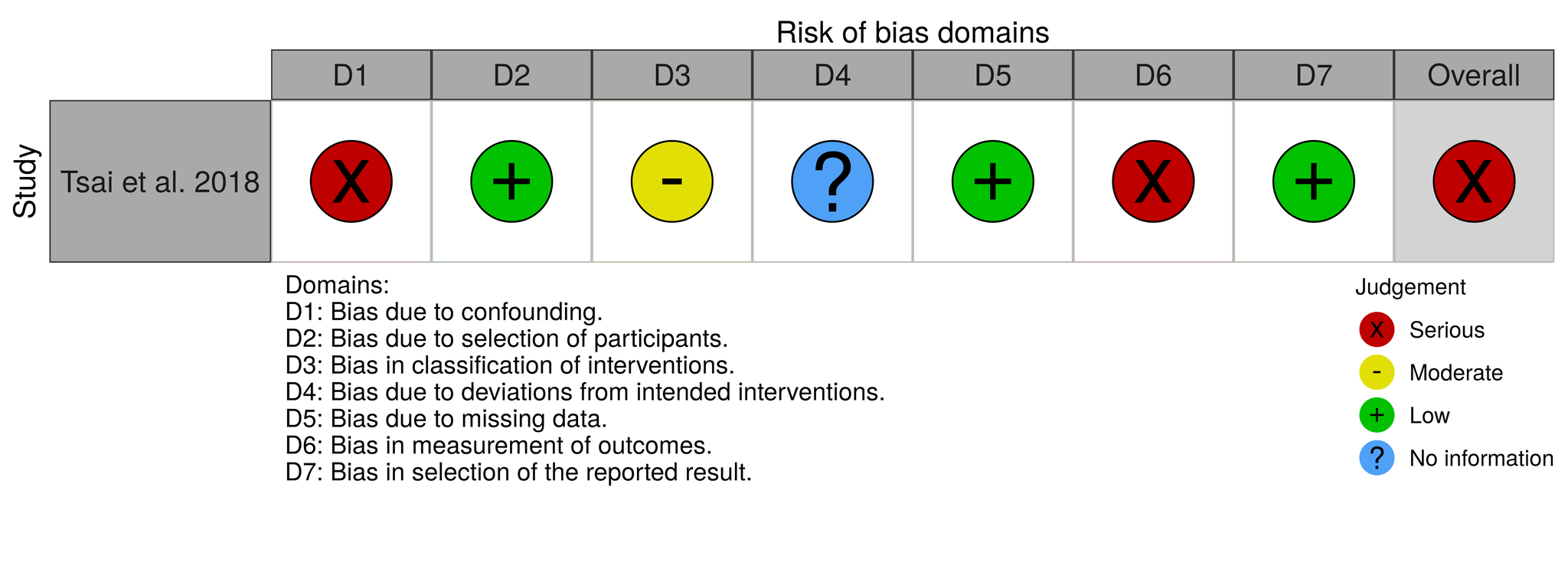


**Figure 9:** Risk of bias assessments on psychological stress from non-randomized controlled studies using the Cochrane *Risk of Bias In Non-randomized Studies of Interventions* Tool (ROBINS-I) - study level

**4. Visual representations of critically appraising results on the utilization of pregnancy-related and mental health care and services**

**
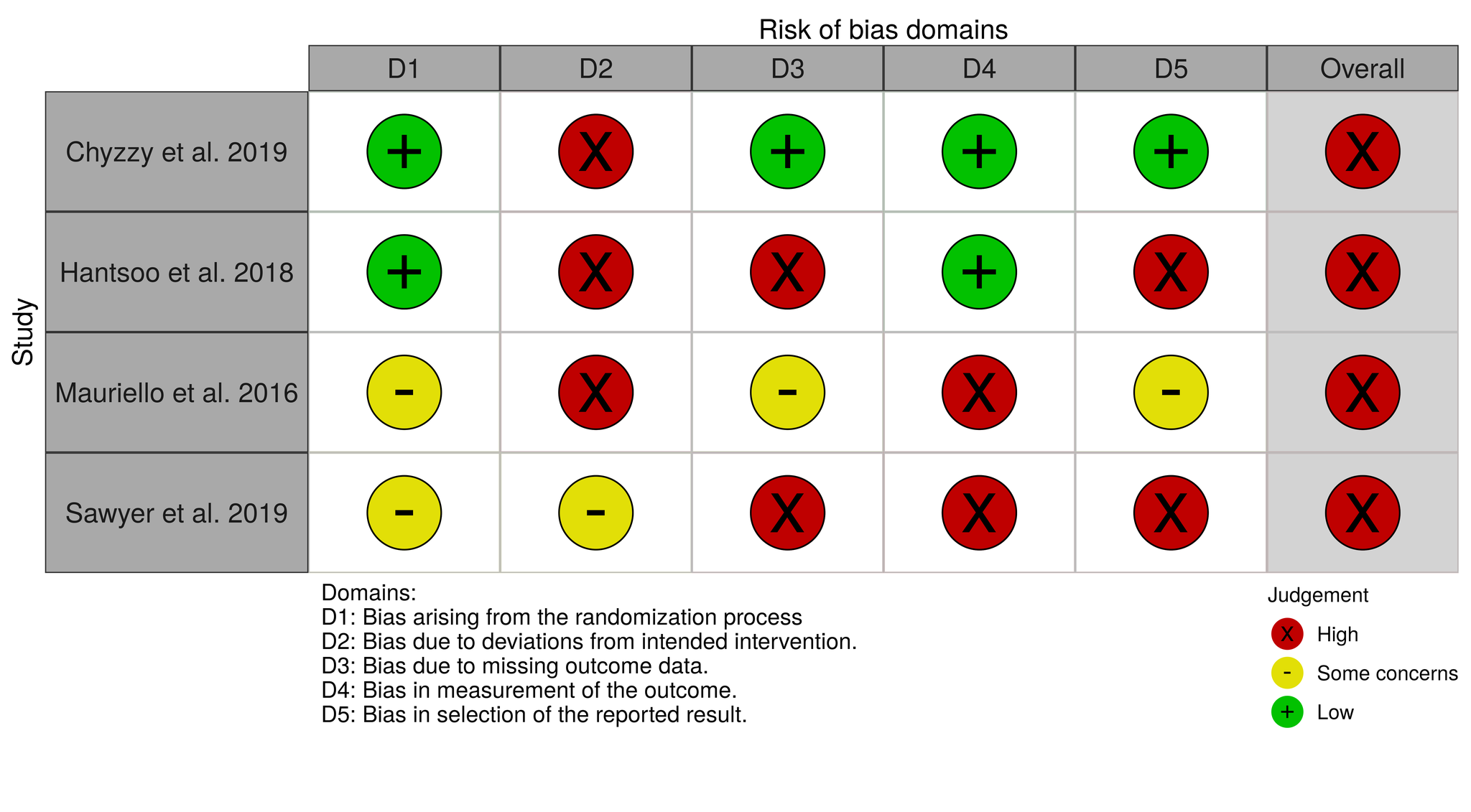
**

**Figure 10**: Risk of bias assessments on utilization of pregnancy-related and mental health care and services from randomized controlled trials of interventions using the Cochrane *Risk of Bias 2.0* tool (ROB 2.0) - study level

**
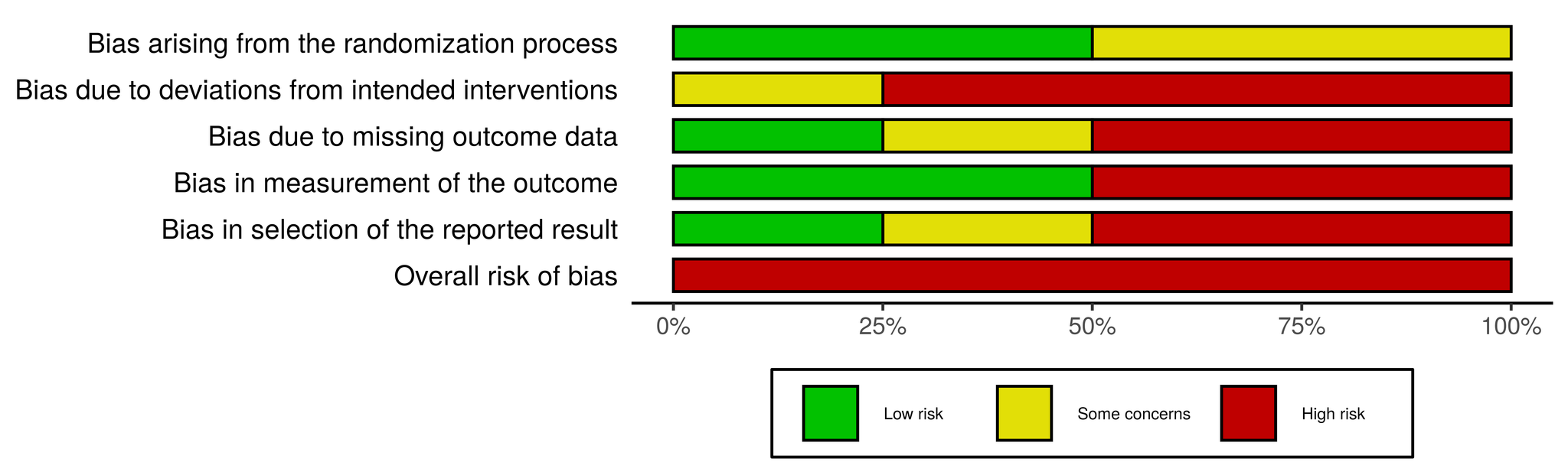
**

**Figure 11**: Risk of bias assessments on utilization of pregnancy-related and mental health care and services from randomized controlled trials of interventions using the Cochrane *Risk of Bias 2.0* tool (ROB 2.0) - across studies
