# Supplementary material for: Mobile interventions targeting common mental disorders among pregnant and postpartum women: An equity-focused systematic review
Source: PLoS One. 2021 Oct 29;16(10):e0259474. doi: 10.1371/journal.pone.0259474 (PMC8555821; doi:10.1371/journal.pone.0259474)
Supplement: S7 File — (DOCX) [file pone.0259474.s007.docx]

**Mobile interventions targeting common mental disorders among pregnant and postpartum women: An equity-focused systematic review**

**Appendix VII: GRADE Evidence Profiles**

**Table I: GRADE Evidence profiles: Outcomes of mobile interventions targeting the *prevention* of mental health disorders**

| **Certainty assessment** | | | | | | | **№ of patients** | | **Effect** | | **Certainty** | **Importance** |
| --- | --- | --- | --- | --- | --- | --- | --- | --- | --- | --- | --- | --- |
| **№ of studies** | **Study design** | **Risk of bias** | **Inconsistency** | **Indirectness** | **Imprecision** | **Other considerations** | **Intervention** | **Control** | **Relative**  **(95% CI)** | **Absolute**  **(95% CI)** |  |  |
| **Outcome: Severity of mental health symptoms** | | | | | | | | | | | | |
| **Severity of depression symptoms-short term (Study IDs: Cheng 2016+Chyzzy 2019; Chan 2019; Gong 2020; Lee 2017; Shorey 2019; Shorey 2017) [assessed with: Edinburgh Postnatal Depression Scale (EPDS)]** | | | | | | | | | | | | |
| 2 | randomised trials | very serious ^a,b^ | not serious | not serious | serious ^c^ | none | 78 | 81 | - | MD **3.07 lower**  (4.68 lower to 1.46 lower) | ⨁◯◯◯  VERY LOW | CRITICAL |
| 1 | randomised trials | not serious | not serious | not serious | not serious | none | 330 | 330 | - | MD **0.65 lower**  (1.29 lower to 0 ) | ⨁⨁⨁⨁  HIGH | CRITICAL |
| 1 | Non-  randomised trials | very serious ^d^ | not serious | not serious | not serious | none | 1481 | 1855 | - | MD **1.3 lower**  (1.58 lower to 1.02 lower) | ⨁⨁◯◯  LOW | CRITICAL |
| 1 | Non- randomised trials | very serious ^e^ | not serious | not serious | serious ^c^ | none | 26 | 26 | - | MD **2.68 lower**  (4.86 lower to 0.5 lower) | ⨁◯◯◯  VERY LOW | CRITICAL |
| 1 | randomised trials | not serious | not serious | not serious | serious ^c^ | none | 54 | 57 | - | MD **2.11 lower**  (4 lower to 0.3 lower) | ⨁⨁⨁◯  MODERATE | CRITICAL |
| 1 | randomised trials | not serious | not serious | not serious | serious ^c^ | none | 63 | 62 | - | MD **0.69 lower**  (1.66 lower to 0.29 higher) | ⨁⨁⨁◯  MODERATE | CRITICAL |
| **Severity of anxiety symptoms-short term (Study ID: Chan 2019) [assessed with: The Depression, Anxiety, and Stress Scale (DASS)]** | | | | | | | | | | | | |
| 1 | randomised trials | not serious | not serious | not serious | serious ^f^ | none | 330 | 330 | - | MD **0.01 higher**  (0.3 lower to 0.32 higher) | ⨁⨁⨁◯  MODERATE | CRITICAL |
| **Severity of anxiety symptoms-short term (Study ID: Chyzzy 2019; Shorey 2019) [assessed with: The State-Trait Anxiety Inventory (STAI)]** | | | | | | | | | | | | |
| 1 | randomised trials | serious ^a^ | not serious | not serious | serious ^c^ | none | 17 | 15 | - | MD **2 lower**  (7.71 lower to 3.71 higher) | ⨁⨁◯◯  LOW | CRITICAL |
| 1 | randomised trials | not serious | not serious | not serious | serious ^c^ | none | 52 | 53 | - | MD **2.45 lower**  (9.9 lower to 5 higher) | ⨁⨁⨁◯  MODERATE | CRITICAL |
| **Severity of prenatal anxiety-short term (Study ID: Jareethum 2008) [assessed with: Questionnaire]** | | | | | | | | | | | | |
| 1 | randomised trials | very serious ^g^ | not serious | not serious | serious ^c^ | none | 32 | 29 | - | MD **2.15 lower**  (3.42 lower to 0.88 lower) | ⨁◯◯◯  VERY LOW | CRITICAL |
| **Severity of perinatal anxiety-short term (Study ID: Jareethum 2008) [assessed with: Questionnaire]** | | | | | | | | | | | | |
| 1 | randomised trials | very serious ^g^ | not serious | not serious | serious ^c^ | none | 32 | 29 | - | MD **1.01 lower**  (2.28 lower to 0.26 higher) | ⨁◯◯◯  VERY LOW | CRITICAL |
| **Outcome: Psychological wellbeing and distress** | | | | | | | | | | | | |
| **Psychological stress-short term (Study ID: Chan 2019) [assessed with: The Depression, Anxiety, and Stress Scale (DASS)]** | | | | | | | | | | | | |
| 1 | randomised trials | not serious | not serious | not serious | serious ^f^ | none | 330 | 330 | - | MD **0.07 higher**  (0.35 lower to 0.5 higher) | ⨁⨁⨁◯  MODERATE | CRITICAL |
| **Psychological stress-short term (Study ID: Cheng 2016) [assessed with: The Perceived Stress Scale]** | | | | | | | | | | | | |
| 1 | randomised trials | very serious ^b^ | not serious | not serious | serious ^c^ | none | 61 | 65 | - | MD **3.52 lower**  (4.95 lower to 2.09 lower) | ⨁◯◯◯  VERY LOW | CRITICAL |
| **Psychological stress-short term (Study ID: Tsai 2018) [assessed with: The 36-item Pregnancy Stress Rating Scale (PSRS-36)]** | | | | | | | | | | | | |
| 1 | Non-  randomised trials | very serious ^h^ | not serious | not serious | serious ^c^ | none | 68 | 67 | - | MD **11.12 lower**  (17.19 lower to 5.05 lower) | ⨁◯◯◯  VERY LOW | CRITICAL |
| **Outcome: Changes in occurrence of mental health disorders** | | | | | | | | | | | | |
| **Odds of not having depression post-intervention-short term (Study ID: Gong 2020) [assessed with: Edinburgh Postnatal Depression Scale (EPDS)>9]** | | | | | | | | | | | | |
| 1 | Non- randomised trials | very serious ^d^ | not serious | not serious | not serious | none | 133/1481 (9.0%) | 299/1855 (16.1%) | **OR 0.51**  (0.41 to 0.64) | **72 fewer per 1,000**  (from 88 fewer to 52 fewer) | ⨁⨁◯◯  LOW | CRITICAL |
| **Outcome: Utilization of pregnancy-related and mental health care** | | | | | | | | | | | | |
| **Mean number of healthcare visits-short term (Study ID: Chyzzy 2019) [assessed with: Health Services Utilization Questionnaire]** | | | | | | | | | | | | |
| 1 | randomised trials | serious ^a^ | not serious | not serious | serious ^c^ | none | 17 | 16 | - | MD **5.3 higher**  (6.97 lower to 17.57 higher) | ⨁⨁◯◯  LOW | CRITICAL |
| **Percentage of patients who visited their family physician-short term (Study ID: Chyzzy 2019) [assessed with: Health Service Utilization Questionnaire]** | | | | | | | | | | | | |
| 1 | randomised trials | serious ^a^ | not serious | not serious | serious ^c^ | none | 15/17 (88.2%) | 14/16 (87.5%) | **RR 1.01**  (0.78 to 1.29) | **9 more per 1,000**  (from 192 fewer to 254 more) | ⨁⨁◯◯  LOW | CRITICAL |
| **Percentage of patients who visited their obstetrician-short term (Study ID: Chyzzy 2019) [assessed with: Health Service Utilization Questionnaire]** | | | | | | | | | | | | |
| 1 | randomised trials | serious ^a^ | not serious | not serious | serious ^c^ | none | 3/17 (17.6%) | 5/16 (31.3%) | **RR 0.56**  (0.16 to 1.99) | **137 fewer per 1,000**  (from 263 fewer to 309 more) | ⨁⨁◯◯  LOW | CRITICAL |
| **Percentage of patients who visited their psychologist-short term (Study ID: Chyzzy 2019) [assessed with: Health Service Utilization Questionnaire]** | | | | | | | | | | | | |
| 1 | randomised trials | serious ^a^ | not serious | not serious | serious ^c^ | none | 0/17 (0.0%) | 2/16 (12.5%) | **RR 0.19**  (0.01 to 3.66) | **101 fewer per 1,000**  (from 124 fewer to 333 more) | ⨁⨁◯◯  LOW | CRITICAL |
| **Percentage of patients who visited their psychiatrist-short term (Study ID: Chyzzy 2019) [assessed with: Health Service Utilization Questionnaire]** | | | | | | | | | | | | |
| 1 | randomised trials | serious ^a^ | not serious | not serious | serious ^c^ | none | 1/17 (5.9%) | 2/16 (12.5%) | **RR 0.47**  (0.05 to 4.70) | **66 fewer per 1,000**  (from 119 fewer to 463 more) | ⨁⨁◯◯  LOW | CRITICAL |
| **Number of minutes practicing stress management-short term (Study ID: Mauriello 2016) [assessed with: Questionnaire]** | | | | | | | | | | | | |
| 1 | randomised trials | very serious ^i^ | not serious | not serious | serious ^c^ | none | The mean difference in number of minutes was not significant between groups when adjusting for minutes of stress management at baseline (Intervention N=52 M=45.77 SD 50, Control N=55 M=30.0 SD 29.4; adjusted p=.288) | | | | ⨁◯◯◯  VERY LOW | CRITICAL |
| **Number of minutes practicing stress management-medium term (Study ID: Mauriello 2016) [assessed with: Questionnaire]** | | | | | | | | | | | | |
| 1 | randomised trials | very serious ^i^ | not serious | not serious | serious ^c^ | none | The mean difference in number of minutes was not significant between groups when adjusting for minutes of stress management at baseline (Intervention N=47 M=61.06 SD 54.1, Control N=49 M=52.86 SD 37.3; adjusted p=.559) | | | | ⨁◯◯◯  VERY LOW | CRITICAL |

**CI:** Confidence interval**; MD:** Mean difference**; OR:** Odds ratio**; RR:** Risk ratio

#### **Explanations**

**a**. High risk of bias due to concerns of deviations from assignment to the intervention (Chyzzy 2019)

**b**. High risk of bias due to concerns with randomisation, deviation from assignment to the intended intervention, and measurement of outcomes. Some concerns with selective outcome reporting (Cheng 2016)

**c**. Optimal Information Size (OIS) not reached: Sample < 300

**d**. Serious concerns regarding confounding, missing outcome data and measurement of outcomes (Gong 2020)

**e.** Serious concerns due to confounding and bias in measurements of outcomes (Lee 2017)

**f**. Confidence interval crosses the null value and excludes meaningful values

**g.** High risk of bias due to concerns with deviation from assignment to the intervention and measurement of outcomes. Some concerns with randomisation and selective outcome reporting (Jareethum 2008)

**h**. Serious concerns due to confounding and bias in measurement of outcomes, moderate risk due to the classification of interventions (Tsai 2018)

**i.** High risk of bias due to concerns with deviation from assignment to intended interventions and measurement of outcomes. Some concerns with randomisation, missing outcome data, and selective outcome reporting (Mauriello 2016)

**Table II: GRADE Evidence profiles: Outcomes of mobile interventions targeting the *management* of mental health disorders**

| **Certainty assessment** | | | | | | | **№ of patients** | | **Effect** | | **Certainty** | **Importance** |
| --- | --- | --- | --- | --- | --- | --- | --- | --- | --- | --- | --- | --- |
| **№ of studies** | **Study design** | **Risk of bias** | **Inconsistency** | **Indirectness** | **Imprecision** | **Other considerations** | **Intervention** | **Control** | **Relative**  **(95% CI)** | **Absolute**  **(95% CI)** |  |  |
| **Outcome 1: Severity of mental health symptoms** | | | | | | | | | | | | |
| **Severity of depression symptoms-short term (Study ID: Baumel 2018; Jannati 2020; Prasad 2018+ Sawyer 2019) [assessed with: Edinburgh Postnatal Depression Scale (EPDS)]** | | | | | | | | | | | | |
| 1 | Non- randomised trials | serious ^a^ | not serious | not serious | serious ^b^ | none | 17 | 17 | - | MD 2.82 higher  (0.49 lower to 6.13 higher) | ⨁⨁◯◯  LOW | CRITICAL |
| 1 | randomised trials | very serious ^c^ | not serious | not serious | serious ^b^ | none | 38 | 37 | - | MD 6.87 lower  (7.92 lower to 5.82 lower) | ⨁◯◯◯  VERY LOW | CRITICAL |
| 2 | randomised trials | very serious ^d,e^ | not serious | not serious | serious ^b^ | none | 77 | 77 | - | MD 0.93 lower  (2.08 lower to 0.21 higher) | ⨁◯◯◯  VERY LOW | CRITICAL |
| **Severity of depression symptoms-long term (Study ID: Sawyer 2019) [assessed with: Edinburgh Postnatal Depression Scale (EPDS)]** | | | | | | | | | | | | |
| 1 | randomised trials | very serious ^d^ | not serious | not serious | serious ^b^ | none | Linear regression results showed small significant improvements associated with the intervention compared to the control group (Intervention N=54 M=8.4 95% CI 7.2 to 9.6, control N=57 M=7.2 95% CI 5.9 to 8.3; Group x time p=.001) | | | | ⨁◯◯◯  VERY LOW | CRITICAL |
| **Severity of anxiety symptoms-short term (Study ID: Constant 2014) [assessed with: The Hospital Anxiety and Depression Scale (HADS)]** | | | | | | | | | | | | |
| 1 | randomised trials | very serious ^f^ | not serious | not serious | serious ^b^ | none | 197 | 184 | - | MD 1.3 lower  (2.33 lower to 0.27 lower) | ⨁◯◯◯  VERY LOW | CRITICAL |
| **Outcome: Psychological wellbeing and distress** | | | | | | | | | | | | |
| **Psychological wellbeing-short term (Study ID: Carissoli 2017) [assessed with: The Italian Psychological Wellbeing Questionnaire (PWB)]** | | | | | | | | | | | | |
| 1 | randomised trials | very serious ^g^ | not serious | not serious | serious ^b^ | none | ANOVA analysis showed significant improvement in autonomy associated with the intervention over time (Intervention N=35 M=4.33, control N=43 M=4.42; F=5.725; p<.05), whereas changes in other constructs were not significant, such as environmental mastery (p=.97), personal growth(p=.34), positive relations (p=.73), purpose in life (p=.13), and self-acceptance (p=.19). | | | | ⨁◯◯◯  VERY LOW | CRITICAL |
| **Subjective stress: Intrusive and avoidance-short term (Study ID: Constant 2014) [assessed with: The Revised Impact of Event Scale (IES-R)]** | | | | | | | | | | | | |
| 1 | randomised trials | very serious ^f^ | not serious | not serious | serious ^b^ | none | When adjusting for baseline anxiety, avoidance-based stress was significantly reduced in the intervention group (N=197 M=13.1 SD=7.3) compared to the control group (N=184 M=14.4 SD=7.4); linear regression coefficient B=-1.8; 95% CI -3.2 to -0.4; p=.015. However, changed in intrusive-based stress remained non-significant (B=-1.4; 95% CI -2.9, 0.2; p=.08). | | | | ⨁◯◯◯  VERY LOW | CRITICAL |
| **Biological stress-short term (Study ID: Dennis-Tiwary 2017) [assessed with: Lab cortisol ug/dl]** | | | | | | | | | | | | |
| 1 | randomised trials | serious ^h^ | not serious | not serious | serious ^b^ | none | ANOVA analysis showed a significant reduction in post-interveniton lab cortisol levels associated with receiving the intervention (Intervention N=15, M=-0.05 SD 0.09, control N=14 M=0.01 SD 0.09; F(1,22)=4.96; p=.037) | | | | ⨁⨁◯◯  LOW | CRITICAL |
| **Psychological wellbeing-short term (Study ID: Prasad 2018) [assessed with: The WHOQOL-BREF scale]** | | | | | | | | | | | | |
| 1 | randomised trials | very serious ^e^ | not serious | not serious | serious ^b^ | none | ANOVA results showed significant improvement in psychological wellbeing associated with the intervention across time (Intervention N=23 M=18.13 SD 1.75, control N=20 M=17.90 SD 1.71; F=4.568; p=.039) | | | | ⨁◯◯◯  VERY LOW | CRITICAL |
| **Outcome: Utilization of pregnancy-related and mental health care** | | | | | | | | | | | | |
| **Percentage of patients who had an obstetrician visit that addressed mental health-short term (Study ID: Hantsoo 2018) [assessed with: Electronic Health Records]** | | | | | | | | | | | | |
| 1 | randomised trials | very serious ^i^ | not serious | not serious | serious ^b^ | none | Compared to the control group, women receiving the intervention had more telephone encounters with their providers than addressed mental health (Intervention N=41 M=0.98 SD 1.3, control N=23 M=0.1 SD 4.7; F=6.0, p=.02) | | | | ⨁◯◯◯  VERY LOW | CRITICAL |
| **Percentage of patients who were referred to a mental health specialist-short term (Study ID: Hantsoo 2018) [assessed with: Electronic Health Records]** | | | | | | | | | | | | |
| 1 | randomised trials | very serious ^i^ | not serious | not serious | serious ^b^ | none | 11/41 (26.8%) | 5/23 (21.7%) | RR 1.23  (0.49 to 3.11) | 50 more per 1,000  (from 111 fewer to 459 more) | ⨁◯◯◯  VERY LOW | CRITICAL |
| **Percentage of patients who attended a mental health specialist visit-short term (Study ID: Hantsoo 2018) [assessed with: Electronic Health Records]** | | | | | | | | | | | | |
| 1 | randomised trials | very serious ^i^ | not serious | not serious | serious ^b^ | none | 3/11 (27.3%) | 1/5 (20.0%) | RR 1.36  (0.17 to 10.09) | 72 more per 1,000  (from 166 fewer to 1,000 more) | ⨁◯◯◯  VERY LOW | CRITICAL |
| **Percentage of patients who visited their family physician 2 times or more in the past 6 months-long term (Study ID: Sawyer 2019) [assessed with: Questionnaire]** | | | | | | | | | | | | |
| 1 | randomised trials | very serious ^j^ | not serious | not serious | serious ^b^ | none | 42/54 (77.8%) | 36/57 (63.2%) | RR 1.23  (0.96 to 1.57) | 145 more per 1,000  (from 25 fewer to 360 more) | ⨁◯◯◯  VERY LOW | CRITICAL |
| **Percentage of patients who visited the emergency department 2 times of more in the past 6 months-long term (Study ID: Sawyer 2019) [assessed with: Questionnaire]** | | | | | | | | | | | | |
| 1 | randomised trials | very serious ^j^ | not serious | not serious | serious ^b^ | none | Results show that receiving the intervention was associated with an increase in the percentage of women who visited the emergency department 2 times or more in the past 6 months (Intervention 8/54=15%, control 2/54=4%; RR=4.00, 95% CI 0.89, 17.98; adjusted p=.04) | | | | ⨁◯◯◯  VERY LOW | CRITICAL |
| **Percentage of patients who used online pregnancy resources in the past 6 months-long term (Study ID: Sawyer 2019) [assessed with: Questionnaire]** | | | | | | | | | | | | |
| 1 | randomised trials | very serious ^j^ | not serious | not serious | serious ^b^ | none | Results show that receiving the intervention was associated with an increase in the percentage of women who used pregnancy-related online resources (Intervention 22/54=41%, control 12/56=21%; RR=1.90, 95% CI 1.04, 3.44; p=.03) | | | | ⨁◯◯◯  VERY LOW | CRITICAL |

**CI:** Confidence interval; **MD:** Mean difference; **RR:** Risk ratio

#### **Explanations**

**a**. Concerns of moderate bias due to confounding. No information regarding missing outcome data and deviation from assignment to intended intervention (Baumel 2018)

**b**. Optimal Information Size (OIS) not reached: Sample < 300

**c**. High concerns of bias due to randomisation, deviation from assignment to intended intervention, and measurement of outcomes. Some concerns with selective outcome reporting (Jannati 2020)

**d**. High concerns of bias due to missing outcome data and measurement of outcomes. Some concerns regarding randomisation and deviation from assignment to intended intervention (Sawyer 2019)

**e**. High concerns of risk of bias due to deviation from assignment to intended interventions, missing outcome data, and measurement of outcomes. Some concerns regarding randomisation (Prasad 2018)

**f**. High concerns of risk of bias due to missing outcome data and measurement of outcomes. Some concerns regarding deviation from assignment to intended intervention and selective outcome reporting (Constant 2014)

**g**. High concerns of risk of bias due to deviation from assignment to intended intervention, missing outcome data, and measurement of outcomes. Some concerns regarding randomisation and selective outcome reporting (Carissoli 2017)

h. High concerns of selective outcome reporting, some concerns regarding randomisation and deviation from assignment to intended interventions (Dennis-Tiwary 2017b)

i. High concerns of risk of bias due to deviation from assignment to intended intervention, missing outcome data, and selective outcome reporting (Hantsoo 2018)

j. High concerns of risk of bias due to missing outcome data, measurement of outcomes, and selective outcome reporting. Some concerns regarding randomisation and deviation from assignment to intended interventions (Sawyer 2019)
