# Supplementary material for: Mobile interventions targeting common mental disorders among pregnant and postpartum women: An equity-focused systematic review
Source: PLoS One. 2021 Oct 29;16(10):e0259474. doi: 10.1371/journal.pone.0259474 (PMC8555821; doi:10.1371/journal.pone.0259474)
Supplement: S8 File — (DOCX) [file pone.0259474.s008.docx]

**Mobile interventions targeting common mental disorders among pregnant and postpartum women: An equity-focused systematic review**

**Appendix VIII**: **Compartmentalized (outcome x PROGRESS+) table**

| **PROGRESS+** | **Severity of mental health symptoms** | **Psychological wellbeing and distress** | **Changes in occurrence of mental health illnesses** | **Utilization of pregnancy and mental health care** |
| --- | --- | --- | --- | --- |
| **P**lace of residence | N/A | N/A | N/A | N/A |
| **R**ace, ethnicity, culture | **Prevention interventions**  Receiving antenatal prevention interventions significantly reduced depression severity among **Chinese** women (MD=−0.65; 95% CI −1.29 to 0.00; p=.049) (Chan et al. 2019)  Receiving antenatal SMS prevention interventions significantly reduced depression among **Chinese** women (MD=-1.30; t=9.03; p<.001) (Gong et al. 2020)  Receiving antenatal prevention interventions did **not** significantly reduce the severity of anxiety symptoms among **Chinese** women (p=.94) (Chan et al. 2019)  Receiving antenatal SMS prevention interventions reduced prenatal anxiety among **Thai** women (MD=-1.01; 95% CI: -3.42, -0.88; p=.002) (Jareethum et al. 2008)  Receiving antenatal SMS prevention interventions did not significantly reduce perinatal anxiety among **Thai** women (p=.12) (Jareethum et al. 2008)  Postnatal prevention interventions were associated with decreased severity of depression among **South Korean** women (MD=-2.68; 95% CI: -4.86 to -0.5; p=.02) (Lee and Kim 2017)  Compared to our pooled results, perinatal prevention interventions were more effective in decreasing the severity of depression symptoms among **Taiwanese** women (MD=-3.70; 95% CI -5.27 to -2.13; p<.001) (Cheng et al. 2016)  The difference in depression severity was non-significant before and after adjusting for **ethnicity** (p=.23 and p=.16, respectively) (Shorey et al. 2017)  **Management interventions**  Postnatal CBT interventions significantly reduced depression severity among **Persian** women (MD=-6.87; 95% CI -7.92 to -5.82; p<.001) (Jannati et al. 2020) | **Prevention interventions**  Antenatal prevention interventions showed significant improvement in psychological stress among **Taiwanese** women (MD=-11.12; 95% CI -17.19, -5.05; p<.001) (Tsai et al. 2018)  Perinatal preventative interventions significantly improved **Taiwanese** women's stress levels (MD=-3.52; 95% CI -4.95 to -2.09; p<.001( (Cheng et al. 2016)  Receiving antenatal prevention interventions did **not** significantly reduce stress levels among **Chinese** women (p=.74) (Chan et al. 2019)  **Management interventions**  Perinatal management interventions significantly improved **Italian** women's sense of autonomy as a construct of psychological wellbeing (MD=-0.09; ANOVA Group x time p<.05) (Carissoli et al. 2017)  Perinatal management interventions did not significantly improve Italian women’s environmental mastery as a construct of psychological wellbeing (p=.78) (Carissoli et al. 2017)  Perinatal management interventions did not significantly improve Italian women’s personal growth as a construct of psychological wellbeing (p=.88) (Carissoli et al. 2017)  Perinatal management interventions did not significantly improve Italian women’s positive relations as a construct of psychological wellbeing (p=.60) (Carissoli et al. 2017)  Perinatal management interventions did not significantly improve Italian women’s purpose in life as a construct of psychological wellbeing (p=.59) (Carissoli et al. 2017)  Perinatal management interventions did not significantly improve Italian women’s self-acceptance as a construct of psychological wellbeing (p=.93) (Carissoli et al. 2017) | **Prevention interventions**  Receiving antenatal SMS prevention interventions significantly reduced the occurrence of depression among **Chinese** women (OR=1.95; 95% CI 1.57 to 2.42; p<.001) (Gong et al. 2020) | **Management interventions**  Antenatal management interventions promoted addressing mental health during routine phone call providers among pregnant women from racial minorities (African American and Latino) (F=6.0; p=.02) (Hantsoo et al. 2018)  Antenatal management interventions did not increase the rate of referral to mental health specialists among pregnant women from racial minorities (African American and Latino) (p=.65) (Hantsoo et al. 2018)  Antenatal management interventions did not increase the rate of visits to mental health specialists among pregnant women from racial minorities (African American and Latino) (p=.76) (Hantsoo et al. 2018) |
| **O**ccupation | **Prevention interventions**  The difference in depression severity was non-significant before and after adjusting for employment status (p=.23 and p=.16, respectively) (Shorey et al. 2017) | N/A | **Prevention interventions**  When adjusting for work intensity among other variables, receiving antenatal SMS prevention interventions was more significantly effective in reducing the occurrence of depression among Chinese women than before adjusting (Unadjusted OR=1.95, adjusted OR=2.04; 95% CI 1.62 to 2.58; p<.001) (Gong et al. 2020) | N/A |
| **G**ender, sex | N/A | N/A | N/A | N/A |
| **R**eligion | N/A | N/A | N/A | N/A |
| **E**ducation | **Prevention interventions**  The difference in depression severity was non-significant before and after adjusting for education (p=.23 and p=.16, respectively) (Shorey et al. 2017)  **Management interventions**  The difference in depression severity was not significant before adjusting for education among other variables, and only showed small significant improvements after adjusting for it (MD=1.2; Group x time p=.001) (Sawyer et al. 2019)  The effectiveness of postnatal CBT interventions was not associated with women’s education (Post-intervention correlation p=.44 and p=.89 for the intervention and control groups, respectively) (Jannati et al. 2020)  Post-abortion SMS interventions were more effective among those who had less than a high school education compared to those who had high school education (p=0.047) (Constant et al. 2014) | N/A | **Prevention interventions**  When adjusting for education level among other variables, receiving antenatal SMS prevention interventions was more significantly effective in reducing the occurrence of depression among Chinese women than before adjusting (Unadjusted OR=1.95, adjusted OR=2.04; 95% CI 1.62 to 2.58; p<.001) (Gong et al. 2020) | N/A |
| **S**ocioeconomic status | **Management interventions**  The effectiveness of postnatal CBT interventions was not associated with women’s income level (Post-intervention correlation p=.55 and p=.79 for the intervention and control groups, respectively) (Jannati et al. 2020) | N/A | **Prevention interventions**  When adjusting for monthly income among other variables, receiving antenatal SMS prevention interventions was more significantly effective in reducing the occurrence of depression among Chinese women than before adjusting (Unadjusted OR=1.95, adjusted OR=2.04; 95% CI 1.62 to 2.58; p<.001) (Gong et al. 2020) | **Management interventions**  Antenatal management interventions promoted mentioning mental health during routine Obstetrician visits among pregnant women with low-income status (F=6.0; p=.02) (Hantsoo et al. 2018)  Antenatal management interventions did not increase the rate of referral to mental health specialists among pregnant women with low income status (p=.65) (Hantsoo et al. 2018)  Antenatal management interventions did not increase the rate of visits to mental health specialists among pregnant women with low income status (p=.76) (Hantsoo et al. 2018) |
| **S**ocial capital | **Prevention interventions**  The difference in depression severity was not significant at 1 month follow-up before and after adjusting for marital status among other variables (p=.25 and p=.23, respectively) (Shorey et al. 2019)  The difference in depression severity was significant at 3 months follow-up before and after adjusting for marital status among other variables (Unadjusted MD=-1.77; 95% CI -3.5 to 0.0;p=.04; adjusted MD=-2.11; 95% CI -4.0 to -0.3; p=.03) (Shorey et al. 2019)  The difference in the severity of anxiety was not significant at 1 month follow-up before and after adjusting for marital status among other variables (p=.31 and p=.52, respectively) (Shorey et al. 2019) | N/A | **Prevention interventions**  When adjusting for marital status among other variables, receiving antenatal SMS prevention interventions was more significantly effective in reducing the occurrence of depression among Chinese women than before adjusting (Unadjusted OR=1.95, adjusted OR=2.04; 95% CI 1.62 to 2.58; p<.001) (Gong et al. 2020) | N/A |
| + Age | **Prevention interventions**  The difference in depression severity was non-significant before and after adjusting for age (p=.23 and p=.16, respectively) (Shorey et al. 2017)  The difference in depression severity was not significant at 1 month follow-up before and after adjusting for age among other variables (p=.25 and p=.23, respectively) (Shorey et al. 2019)  The difference in depression severity was significant at 3 months follow-up before and after adjusting for age among other variables (Unadjusted MD=-1.77; 95% CI -3.5 to 0.0; p=.04; adjusted MD=-2.11; 95% CI -4.0 to -0.3; p=.03) (Shorey et al. 2019)  The difference in the severity of anxiety was not significant at 1 month follow-up before and after adjusting for age among other variables (p=.31 and p=.52, respectively) (Shorey et al. 2019)  Compared to the significant difference of our pooled result, receiving perinatal peer-support interventions did not significantly improve depression severity among pregnant adolescents (p=.11) (Chyzzy 2019)  Receiving perinatal peer-support interventions did not significantly improve the severity of anxiety symptoms among pregnant adolescents (p=.60) (Chyzzy 2019)  **Management interventions**  The difference in depression severity was not significant before adjusting for age among other variables, and only showed small significant improvements after adjusting for it (MD=1.2; Group x time p=.001) (Sawyer et al. 2019)  The effectiveness of postnatal CBT interventions was not associated with women’s age (Post-intervention correlation p=.87 and p=.85 for the intervention and control groups, respectively) (Jannati et al. 2020) | N/A | **Prevention interventions**  When adjusting for age among other variables, receiving antenatal SMS prevention interventions was more significantly effective in reducing the occurrence of depression among Chinese women than before adjusting (Unadjusted OR=1.95, adjusted OR=2.04; 95% CI 1.62 to 2.58; p<.001) (Gong et al. 2020) | **Prevention interventions**  Receiving perinatal peer-support interventions did not significantly improve the mean number of healthcare visits among pregnant adolescents (p=.41) (Chyzzy 2019)  Receiving perinatal peer-support interventions did not significantly impact the percentage of pregnant adolescents visiting their family physician (p=.95) (Chyzzy 2019)  Receiving perinatal peer-support interventions did not significantly impact the percentage of pregnant adolescents visiting their obstetrician (p=.37) (Chyzzy 2019)  Receiving perinatal peer-support interventions did not significantly impact the percentage of pregnant adolescents visiting their psychologist (p=.26) (Chyzzy. 2019)  Receiving perinatal peer-support interventions did not significantly impact the percentage of pregnant adolescents visiting their psychiatrist (p=.52) (Chyzzy 2019) |
| **+** Disability | N/A | N/A | N/A | N/A |
| **+** Time-  dependent:  Being primiparous | **Prevention interventions**  Postnatal prevention interventions were associated with decreased severity of depression among primiparous women (MD=-2.68; 95% CI: -4.86 to -0.5; p=.02) (Lee and Kim 2017)  Receiving antenatal prevention interventions significantly reduced depression severity among first-time mothers (MD=−0.65; 95% CI −1.29 to 0.00; p=.049) (Chan et al. 2019)  Receiving antenatal prevention interventions did not significantly reduce the severity of anxiety symptoms among first-time mothers (p=.94) (Chan et al. 2019) | **Prevention interventions**  Receiving antenatal prevention interventions did not significantly reduce stress levels among primiparous mothers (p=.74) (Chan et al. 2019)  **Management interventions**  Perinatal management interventions significantly improved primiparous women's sense of autonomy as a construct of psychological wellbeing (MD=-0.09; ANOVA Group x time p<.05) (Carissoli et al. 2017)  Perinatal management interventions did not significantly improve primiparous women’s environmental mastery as a construct of psychological wellbeing (p=.78) (Carissoli et al. 2017)  Perinatal management interventions did not significantly improve primiparous women’s personal growth as a construct of psychological wellbeing (p=.88) (Carissoli et al. 2017)  Perinatal management interventions did not significantly improve primiparous women’s positive relations as a construct of psychological wellbeing (p=.60) (Carissoli et al. 2017)  Perinatal management interventions did not significantly improve primiparous women’s purpose in life as a construct of psychological wellbeing (p=.59) (Carissoli et al. 2017)  Perinatal management interventions did not significantly improve primiparous women’s self-acceptance as a construct of psychological wellbeing (p=.93) (Carissoli et al. 2017) | N/A | N/A |
| **+** Time-  dependent: IPV | N/A | N/A | N/A | N/A |
